# Fall Risk Awareness and Safety Precautions Taken by Older Community-Dwelling Women and Men—A Qualitative Study Using Focus Group Discussions

**DOI:** 10.1371/journal.pone.0119630

**Published:** 2015-03-17

**Authors:** Petra Pohl, Marlene Sandlund, Christina Ahlgren, Birgitta Bergvall-Kåreborn, Lillemor Lundin-Olsson, Anita Melander Wikman

**Affiliations:** 1 Department of Community Medicine and Rehabilitation, Physiotherapy, Umeå University, 90187 Umeå, Sweden; 2 Department of Business Administration, Technology and Social Sciences, Luleå University of Technology, 97187 Luleå, Sweden; 3 Department of Health Sciences, Division of Health and Rehabilitation, Luleå University of Technology, 97187 Luleå, Sweden; David Geffen School of Medicine, UNITED STATES

## Abstract

**Introduction:**

Daily life requires frequent estimations of the risk of falling and the ability to avoid a fall. The objective of this study was to explore older women’s and men’s understanding of fall risk and their experiences with safety precautions taken to prevent falls.

**Methods:**

A qualitative study with focus group discussions was conducted. Eighteen community-dwelling people [10 women and 8 men] with and without a history of falls were purposively recruited. Participants were divided into two groups, and each group met four times. A participatory and appreciative action and reflection approach was used to guide the discussions. All discussions were audio recorded and transcribed verbatim. Data were analysed by qualitative content analysis, and categories were determined inductively.

**Findings:**

Three categories describing the process of becoming aware of fall risks in everyday life were identified: 1] Facing various feelings, 2] Recognizing one’s fall risk, and 3] Taking precautions. Each category comprised several subcategories. The comprehensive theme derived from the categories was “Safety precautions through fall risk awareness”. Three strategies of ignoring [continuing a risky activity], gaining insight [realizing the danger in a certain situation], and anticipating [thinking ahead and acting in advance] were related to all choices of actions and could fluctuate in the same person in different contexts.

**Conclusions:**

The fall risk awareness process might be initiated for various reasons and can involve different feelings and precautions as well as different strategies. This finding highlights that there are many possible channels to reach older people with information about fall risk and fall prevention, including the media and their peers. The findings offer a deeper understanding of older peoples’ conceptualizations about fall risk awareness and make an important contribution to the development and implementation of fall prevention programmes.

## Introduction

Falls are the leading cause of injury and death among older adults [1–2] and can lead to a negative spiral of inactivity and decline that put older people closer to or below the critical ‘thresholds’ of performance necessary for everyday activities [3]. Apart from injuries, a fear of falling is a common consequence of falls [4]. Many fall risk factors have been identified in community-dwelling people, including previous falls, old age, female gender, gait and balance impairments, visual impairments, certain diseases and medications, and home hazards [1]. Most falls occur in the home or immediate home surroundings and generally occur on level surfaces during routine activities of daily living [5]. There is a dynamic interaction between environmental conditions and the individuals’ behaviour, and many falls occur when a person fails to avoid hazards or when the environmental demands are excessive in relation to the physical abilities of the individual [6]. Daily life requires frequent estimations of the risk of falling and the ability to avoid a fall, but finding the right balance between risk taking and risk avoidance has been found to be difficult for many older people [7].

Given the serious consequences of falls, it is essential to take a preventive approach. Our clinical experience suggests that older people spontaneously take precautions to avoid falls, e.g., not climbing on stools when reaching for something from a high cabinet. These serve as protective measures against falls, but some older people develop a self-imposed restriction of ordinary activities even without any real danger of falling [8]. Still, there are probably many precautions that older people can take to prevent falls without requiring far-reaching adjustments to either their behaviour or their home environment. There is also strong evidence that multifactorial interventions and specific exercises can reduce the risk of falls [9], but adherence to fall prevention exercises is often poor [10–11]. In order to encourage older people to better manage their day-to-day risk of falling, a self-management approach can be used [12]. Self-management programmes often include components to empower people, to develop problem-solving skills, and to plan appropriate actions [13]. To optimize the conditions for self-management in relation to fall prevention, there is a need to identify what people are actually doing to reduce their fall risk in their daily lives, and what changes they are prepared to make [14]. By including older people in the discussions, we hope to identify and broaden the understanding about their own choices in everyday life that can help them to avoid falling, what brings on these behaviours, and the processes surrounding their decisions. A better knowledge of how fall risk awareness emerges and related attitudes could help when developing and implementing fall preventive strategies and intervention programmes for older community-dwelling people.

The aim of this study was to explore older women’s and men’s understanding of fall risk and their experiences with safety actions taken for preventing falls.

## Methods

We conducted a qualitative study based on focus group discussions. This approach was justified because the aim of the study was to understand and explore older people’s behaviours as well as their cultures and social lives. The focus group discussions have the potential to bring out new information through the continuous exchange of experiences by triggering new thoughts and associations [15–16]. The research team consisted of experts from the fields of physiotherapy, informatics, e-health, and gender studies. To guide the discussions, a participatory and appreciative action and reflection [PAAR] approach was adopted [17]. By using an appreciative inquiry and reflection approach based on positive psychology, PAAR adds a positive resource of inspiration instead of focusing on problems. The PAAR approach allows the researchers to stimulate a process of reflection so that the participants can learn from each other and build upon the positive aspects of their lives [18–19].

### Setting and participants

This qualitative study constituted the initial part of a larger participatory research project with the overall aim of developing an application for smartphones and tablet devices that uses evidence-based exercises to prevent falls. It was predetermined to recruit 18 community-dwelling older women and men in the study. Recruitment took place in seven senior citizen associations as a purposive sampling in September and October 2012 in Umeå, a university city in northern Sweden (latitude 63°N) with distinct summer and winter seasons. Selection criteria were being at least 70 years of age, the ability to speak and understand Swedish fluently, and having a variation of background variables regarding education level, marital status, previous occupation, history of falls, and exercising. In order to reflect the overall population regarding falls, it was decided that 30% of the participants should have experiences from at least one fall in the previous 12 months. A fall was defined as an unexpected event in which a person comes to rest on the ground or floor. Thirty-eight people volunteered to participate and were interviewed by telephone using a structured interview guide. The habitual level of physical activity was estimated on three levels [low, medium, or high] with the International Physical Activity Questionnaire [20]. After completion of the telephone interviews, 18 people were carefully selected based on the criteria to represent a wide variety of experiences and were invited to participate. Three invited individuals declined to participate because they could not attend the first meeting, which was mandatory due to important information being discussed and the need to create a positive atmosphere in the group as a foundation for the coming developments. A fourth individual declined to participate because her best friend had not been selected. Four other people with similar experiences were invited, and these accepted to participate. Informed consent was obtained from the study participants prior to the focus group discussions. No participant dropped out during the study. Characteristics of the participants are shown in Table 1. Two married couples were included, and four people lived alone. The majority of the participants were former skilled white-collar workers and were, in general, fairly physically active. The participants were between 70 and 80 years old with a mean age of 74.6 ± 3.5 years. Ten study participants were female and 8 were male.

**Table 1:** Background information on the 18 participants.

Participant	Focus group	Age	Sex F/M	Living with spouse Yes/No	Falls previous year Yes/No	Physical activity level, intensity
1	1	71	F	Yes	Yes	High
2	1	70	F	Yes	No	Low
3	1	80	F	No	Yes	Low
4	1	75	F	No	No	High
5	1	74	F	Yes	No	High
6	2	74	F	Yes	Yes	Moderate
7	2	76	F	No	No	High
8	2	71	F	Yes	No	High
9	2	73	F	Yes	No	Low
10	2	70	F	Yes	No	High
11	1	79	M	Yes	Yes	Moderate
12	1	79	M	Yes	No	Moderate
13	1	70	M	Yes	No	High
14	1	75	M	Yes	No	High
15	2	72	M	Yes	Yes	Low
16	2	80	M	No	No	Moderate
17	2	76	M	Yes	No	Low
18	2	78	M	Yes	Yes	High

F = Female; M = Male.

Levels of habitual physical activity were categorised as *High* = Vigorous-intensity activity on at least 3 days/week; *Moderate* = 3 or more days/week of vigorous activity of at least 20 minutes per day OR 5 or more days of moderate-intensity activity or walking of at least 30 minutes per day OR 5 or more days of any combination of walking, moderate-intensity or vigorous intensity activities; *Low* = less than moderate

### Ethics Statement

The study was approved by the Regional Ethical Review Board in Umeå, Sweden (Dnr. 2012-170-31 M). All participants provided informed written consent.

### Data collection

Based on the recommendations to use 4–12 people in a focus group [21], the 18 participants were divided into two groups of nine participants in each group prior to the first focus group session. Eight sessions—four sessions per group—were held in a spacey common room at a community centre once a month between October 2012 and January 2013. Each session lasted for 150 minutes, including a short coffee break. Each session had an overall aim of discussing experiences from fall risks in everyday life as well as interests and attitudes about physical activity in general. The specific topics of fall risk awareness and safety strategies were the main focus of these sessions at all times (Table 2).

**Table 2:** Topic guide for the focus group discussions.

Focus group sessions	Topics
October, 2012	Presentation of the larger project, the researchers, and the participants. Discuss the personal meaning of the concepts ‘joy of movement’ and ‘balance’.
November, 2012	Falls and consequences—what do you do to avoid them?
December, 2012	How can new technology inspire you to be physically activity?
January, 2013	Identify strategies to protect from falls in everyday life, why the strategies were adopted and how the strategies are perceived.

The focus group discussions were led by a skilled moderator in a positive atmosphere in the spirit of PAAR with loose, broad, and open-ended questions in order to encourage the participants to freely speak their minds. The researchers were active in the discussions but cautious about not taking over the discussions. In general, the discussions in the groups were fluent and little steering from the moderator was needed. With the intention to explore gendered patterns in the reasoning about fall risk awareness and/or the choices of safety strategies taken, a more focused approach was used during the fourth session. During this last session, both focus groups were divided into three smaller groups of three participants each to ensure that everyone had their say. Two groups consisted of women only, two groups consisted of men only, and two groups were mixed. The participants were given a discussion guide with the following questions: *What strategies, if any, have you changed in your everyday life due to the ageing process or in order to avoid falling*?, *What brought on those changes*?, and *How did you experience making those changes*? None of the researchers were present during the discussions in the smaller groups, but all discussions were digitally recorded. After 30 minutes of discussion, the participants were collected for a joint discussion within the larger focus group and this was also recorded. A total of eight focus group discussions and six minor group discussions were conducted amounting to 22 hours of data. All focus group discussions were transcribed verbatim.

### Analysis

**Fig 1:**
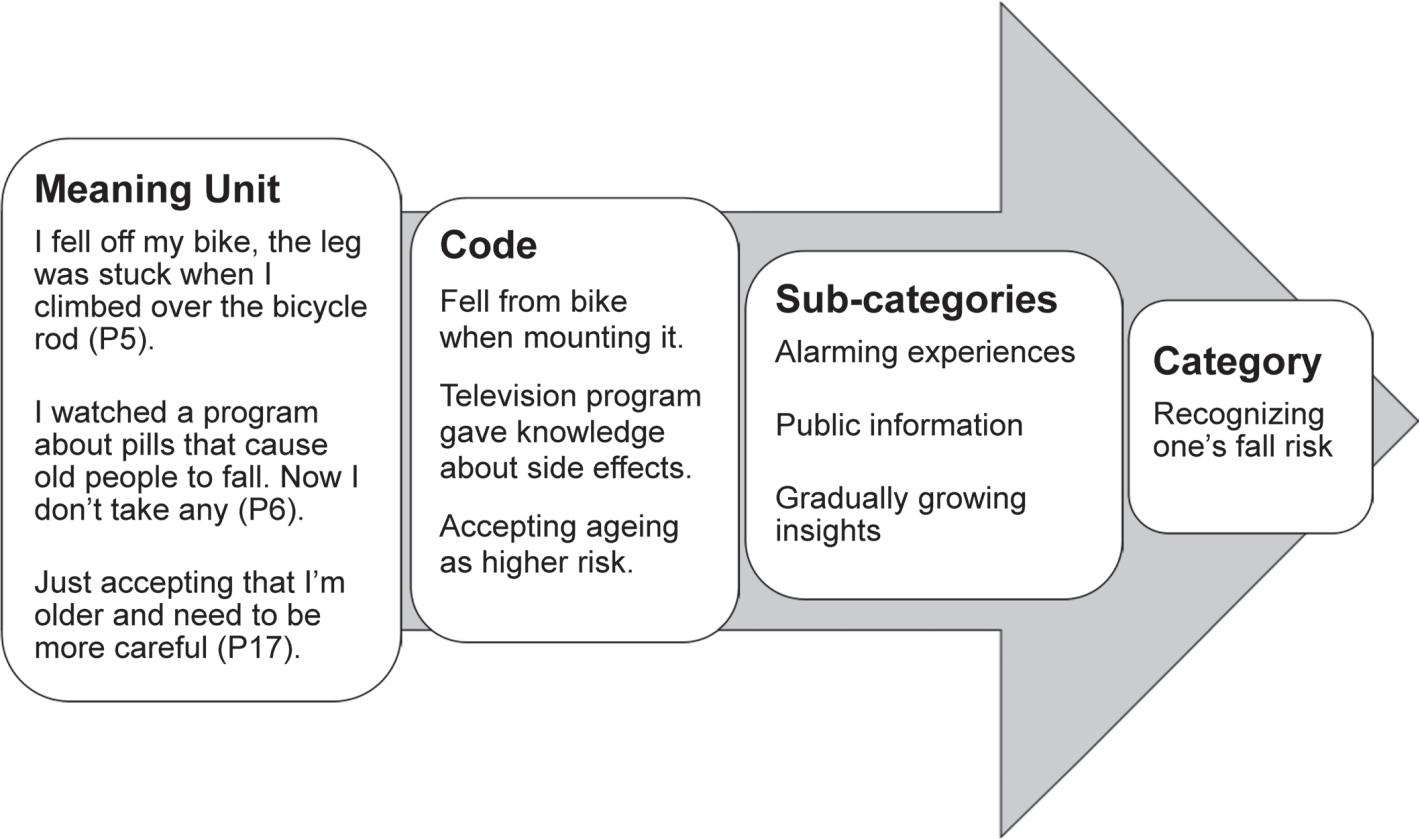
Transforming meaning unit into category. Examples of the transformation process from meaning unit to category using qualitative content analysis.

Qualitative content analysis with an inductive approach was used to analyse the data [22]. This is an appropriate method to highlight similarities and differences in people’s thoughts about their experiences and their actions [23]. All researchers were involved in data analysis. The analysis was performed in several steps. First, the authors independently read the transcripts to get an overall understanding of the participant’s views about, and experiences of, risk awareness. The text was then transferred to the qualitative data software program Open Code 4.01 [24] and divided into meaning units. The meaning units were labelled with codes comprising several words or phrases related to the aim of this study. The codes were organized into preliminary categories and subcategories by two of the authors (PP and MS). To ensure trustworthiness, the categorisation was continuously discussed within the research group until consensus was reached. When uncertainties occurred, the original transcripts were reread by all authors to ensure credibility. An example of the process of transforming a meaning unit into a subcategory and category is shown in Fig. 1.

## Findings

The older participant’s experiences and views relating to fall risk awareness and safety precautions taken in everyday life had several similarities as well as differences. In their stories, we identified three categories describing the process of becoming aware of fall risks in everyday life: *Facing various feelings, Recognizing one’s fall risk*, and *Taking precautions*. The first category captured a spectrum of feelings that are involved in relation to fall risk, the second illustrated possible ways that awareness about fall risk can be initiated, and the third dealt with what older people do themselves to avoid falls. Each category comprised several subcategories. The comprehensive theme that tied the categories together was *Safety precautions through fall risk awareness*.

**Fig 2:**
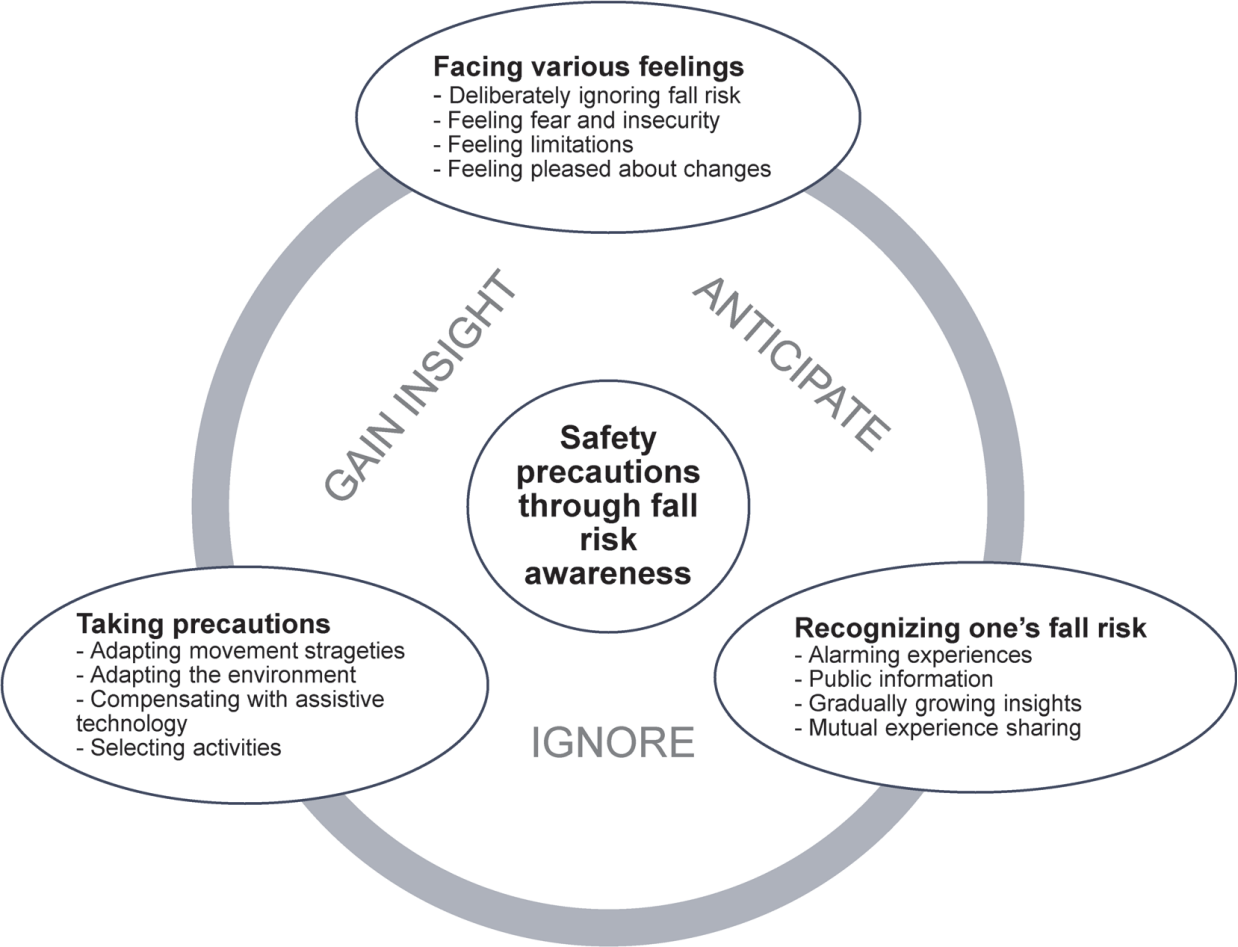
Three categories and one overall theme. A plausible model for development of safety precautions through fall risk awareness and related feelings in older community-dwelling women and men. Ignore, Gain insight and Anticipate are present throughout the intire process of becoming aware of fall risk.

In all three categories, the participant’s awareness processes and the actions they took also fluctuated between the three strategies of *ignoring* (continuing a risky activity), *gaining insight* (realizing the danger in a certain situation), and *anticipating* (thinking ahead and acting in advance). These three strategies were present to different degrees and were either conscious or unconscious. They permeated all of the choices that were made and could vary within the same person in different contexts. The theme, categories, and subcategories are described below and are illustrated with quotations from the participants. An overview of the results is shown in Fig. 2.

### Facing various feelings

Various feelings were associated with an emerging fall risk awareness. Women and men both expressed various feelings, and there were no clear patterns for either sex. Some feelings were related to a gradual loss of function, others to real incidents, and others to an evolving sense of an overall gratitude and content. The following four subcategories were generated: *Deliberately ignoring fall risk, Feeling fear and insecurity, Feeling limitations*, and *Feeling pleased with changes*.

*Deliberately ignoring fall risk* was expressed in a somewhat rebellious manner. The participants still felt young and wanted to continue with old activities and even try new risky activities. They disliked patronizing comments—especially from their grown children—when continuing with potentially risky activities. One man (Participant (P) 15) described how his grandchildren had inspired him to take up downhill skiing again. The joy he felt when skiing with his grandchildren made him overlook the risks involved with this activity. Making the decision to try the activity and to manage the risks made him proud and self-confident, but his son had been concerned. The man says: *“I hadn’t been downhill skiing for about 30 years, but last year I bought some stuff and it was fun, you know*? *My son seemed to think it was fun, too—so far, anyway”*. One woman (P1) with a professional background of taking care of others had experiences from multiple falls, and now lived a full and active life as retired. She made deliberate, active choices such as climbing up on ordinary chairs when reaching for something up high. She herself did not perceive this as risky. Her daughter’s concern annoyed her, she felt fully capable of looking after herself: *“‘But mom’, my daughter said, ‘remember that you’re 70 years old!’ But I’m probably fitter than she is”*.

*Feeling fear and insecurity* was a common feeling among the participants. Many participants feared the consequences from a fall and the impact on their independence that this might have. One man (P18) described how he had fallen unexpectedly and unglamorously while washing his car and how he had felt old and vulnerable: *“Nowadays I feel that I don’t dare fall, and I feel clumsy. Children often fall, but they don’t get hurt much. But if I fall… well, then bad things will happen. I just don’t dare to fall”*. The fear had several dimensions, including not being able to get up from the floor after a fall, not being able to take care of ill partners, and the fear of pain or the inconvenience a fracture would incur. One woman (P1) with experiences from fractures remembers, *“My life became very difficult. I was alone at home with three dogs and my husband was abroad, and there I was with a broken arm and I was supposed to take care of everything with a plaster cast on my arm and…well, it was really hard!”* A fear of looking ridiculous in public was mentioned, and one would always try to get up as quickly as possible, hopefully unnoticed, after a fall.

*Feeling limitations* described restrictions related to the failing body, especially those related to pain or stiffness. An example was slower reaction times and not being able to reach out for support quickly enough. One woman (P2) compared herself with a statue: “*I inevitably come to think about those statues of Stalin and Lenin that they tore down in the 90s. The statues just stood there and fell over. That’s how it feels when you fall, at least for me.”* Sometimes other people’s concerns limited the participants’ everyday activities. One man (P13), who had retired from a typically masculine occupation, remembered that he was quite agonized when his partner forbade him to shovel snow off the roof. The sudden realization that other people no longer see you as the young and fit person you feel inside was a thorn in his side: *“Maybe they see that I’m starting to stumble, I don’t know. Of course it felt…it came as a shock, frankly. I thought of myself as healthy and fit.”* Somewhat contradictorily, he worried about his partner and told her that she should be more careful. He now wondered if this was because he was the strong man who was supposed to take care of her.

*Feeling pleased with changes* included feelings of gratitude, acceptance of the changes that come with age, and even optimism about the future. Participants were pleased to have grown wiser and recognized that life offered experiences that made them more careful. As one man (P11) says, *“You try to embrace the important things in life and try to make a habit out of it, for instance, by being more careful in certain situations.”* The participants with this attitude did not mourn their loss of function, and they were grateful for others’ concern even when their grown children unexpectedly acted protectively, for instance, when buying them a stepladder for safety.

### Recognizing one’s fall risks

The participants agreed that their reflections about fall risk had increased with age, but how and when the recognition and reflections were initiated varied greatly and led to the generation of the following four subcategories: *Alarming experiences, Public information, Gradually growing insights*, and *Mutual experience sharing*.

*Alarming experiences* reflected a sudden onset of awareness after a specific event, including events that happened to other people. Such events were usually well remembered and included, for example, falls, bicycle accidents, or diagnosis of an illness such as osteoporosis. These events sometimes involved fractures and initiated the process of thinking about hazards and possible consequences from a future fall. One woman (P6) says, “*It took me two broken ribs to realize that, no—I can’t continue like before. There is something I need to change.”*

*Public information* described the media’s—especially television’s—influence on initiating fall risk awareness. A reflection process was initiated if the participants had something to relate the new information to. One woman (P6) with longstanding insomnia had repeatedly tried sleeping pills and often felt dizzy in the morning. With great interest she had watched an interview with a professor of geriatric medicine. She says, “*I heard [doctor’s name] on television. He said that X [a common sleeping pill] was the worst tablet old people could take because then they would fall and break their legs and everything. And I was so upset about this, now I never take any sleeping pills. I’m retired—I can stay awake all night.”*

*Gradually growing insights* included descriptions about how risk awareness can creep up on a person slowly and almost unnoticed. These insights were often associated with slowly emerging changes in sensory functions such as vision or balance impairments. These gradual changes were more frequently described by male participants, and women more often referred to a sudden onset of awareness. In response to a direct question about how the fall risk awareness began, one man (P13) reflects, “*It sneaks up on you!”*

*Mutual experience sharing* described how the participants discovered that a new or deepened awareness of fall risks was achieved through the repeated sessions and mutual discussions with the other participants. By exchanging experiences and knowledge, the participants started to observe their own behaviour as well as the environment around them. They continued to reflect at home, talking to friends, family, and neighbours. From their peers in the project, they were given new ideas about helpful assistive devices and about small changes they could make that would lead to a safer environment. Few participants had given these issues much thought before participating in the project, and they stressed the importance of raising such issues in society more often. One woman (P2) with no history of previous falls said that it made her reflect when listening to one of the other participants telling about the slippery soles of her warm shoes when stepping off a bus onto icy ground in the winter. She turns to the woman and says, *“I have actually thought about it…every time I get off a bus I think about you, and I was just thinking about that: ‘Well, now the soles of my shoes are warmer, now I must be careful.’”*

### Taking precautions

The last of the three categories described the participant’s ideas, solutions, physical adaptations, and resources related to fall risk. It represents the actual changes in body movements or compensation strategies that fall under the following four subcategories: *Adapting movement strategies, Adapting the environment, Compensating with assistive technology*, and *Selecting activities*.

*Adapting movement strategies* described both automatic as well as conscious adaptations. Some adaptations such as a slower gait or decreased step-length when walking on icy ground were not new, but were reinforced. With ageing and less flexible bodies, the participants felt an increasing need for physical support or to alter their body positions during everyday life activities. One widower (P16) who now had the full responsibility for cleaning the house says, *“My knee is so stiff that I have to lie flat on my back to vacuum under the bed.”* An additional need to look down at the feet while walking and to fix the gaze while balancing on one leg was also described.

*Adapting the environment* described safety precautions taken in everyday life, including removing loose carpets, using appropriate and safe footwear and anti-slip shoe devices, leaving a small light on during the night, using an anti-slip mat in the shower, replacing the bath tub with a shower, using a step ladder with a handle when collecting items from a higher level, and changing to spiked bicycle tires in the winter. Anti-slip shoe devices were found to be cheap and good fall protectors during the winter, particularly among women. The men tended to avoid these devices using a variety of excuses. In general men also had a delicate problem in relation to bicycling; they agreed among each other that it had become dangerous to step on and off a rolling men’s bike while balancing on one pedal and tossing the other leg over the frame. One strategy was to use a woman’s bike instead, which was mentioned with embarrassment and accompanied by amused laughter. One man (P12) laughs merrily: “*I’ve got a bike for ladies!”* Another man (P11) replies: “*Yes, the frame is tricky. You have to climb over it somehow”*. Not all participants, however, chose to adapt their environment even though they knew there was a risk of falling.

*Compensating with assistive technology* described different ways to improve safety. Independence could be prolonged with small measures, but the opinions about assistive devices were divided. Women in general seemed to accept aids more easily than men. In order to maintain the appearance of being young and fit, the use of walking aids was often frowned upon. Ordinary crutches could be seen as a threat to a person’s pride or as a necessary evil. One man (P17) with severe knee problems required a long period of reflection before finally accepting crutches. Nordic walking poles were in general popular as balance supports and did not come with as much age-related stigma. The man with knee problems (P17) had been physically limited for a long time and he expressed gratitude about learning how to use a new assistive device that gave him back his independence: *“I have always liked to fix things myself, but now I just can’t manage in the same way anymore, and I have accepted that I’m not 17 or 25 anymore and accepted—or rather learned—how to use the assistive device. It has all come back to me, that feeling of ‘I can manage this myself, I can do it myself!’ I don’t have to shout for the kids, ‘Can you help me with this?’ or anything, I can handle it myself again*.” It was also considered a major safety strategy to bring mobile phones when away from home. One woman (P1) with a history of several fractures says, “*I never go out on long walks nowadays without the mobile phone, it’s a security thing that I can call for help. It actually happened once, I was out when it was all wet and slippery, and nobody knew where I was. I had my car parked deep in the forest and my husband was away on a trip, and no one knew that I was away*.”

*Selecting activities* described different strategies related to choosing activities, avoiding activities, or just carrying on as usual. One woman (P4) explained that she would never dream of climbing up on top of a chair that was placed on a high bench like she used to when she was younger. Some participants described an increasing willingness to ask for help when performing activities they felt were risky, for example, changing the curtains. Couples would cooperate when changing a light bulb or cleaning windows, and it was seen as an advantage to live with a partner. Another active choice was to put the bicycle into storage for the winter, but not all participants were prepared to do this. A fear of falling sometimes made people change their minds after negotiating with themselves about engaging in activities that were perceived as risky. For example, one woman (P1) no longer jumped across ditches in the forest after experiencing a severe fall. Another woman (P2) reflected on possible consequences: *“I was alone, and there was this nesting box hanging from the tree, almost falling down. I thought about adjusting it and went for the ladder and put it against the birch and started to… But then I thought, ‘No, you are not going to do this when you are all by yourself!’ So I didn’t do it! But then I thought, ‘Well, now I’m getting old!’”*

### Safety precautions through fall risk awareness

Fall risk awareness in older community-dwelling women’s and men’s everyday lives involves both diverse feelings and processes that operate more or less outside of conscious awareness as well as conscious choices of behaviours. This comprehensive theme describes the complexity of this awareness. The processes are initiated for various reasons and involve various feelings, and safety precautions such as selecting activities, adapting movement strategies, and making changes in the environment.

## Discussion

We found in our qualitative approach that the experiences of fall risk among community-dwelling people over 70 years of age can be described in the context of three main categories: Facing various feelings, Recognizing one’s fall risk, and Taking precautions. Previous experiences played a large part in judgement and decision-making, and it was also a question of attitude as to what challenges the participants were willing to undertake in relation to the risk of falling, e.g., trying new risky activities or accepting assistive devices.

The way the participants described their experiences of, and behavioural responses to, fall risk might be understood to some extent by risk-as-feelings and risk-as-analysis models. Both models are needed in order to make appropriate decisions to navigate safely in the environment [25]. The risk-as-feelings model refers to a rapid affective reaction and adaptation that is oriented around pleasure and pain. For example, a fear of falling based on previous injurious falls might have an impact on gait, e.g., taking smaller steps on icy ground without reflecting consciously about it. The risk-as-analysis model refers to a logic-based mode of estimating risk, and behaviours are mediated by conscious reflections and appraisals. This is a slower process based on probabilities, for example, deciding to putting away the bicycle during the winter or using anti-slip shoes. It has been proposed that ageing itself might lead to an increased reliance on the risk-as-feelings model for a variety of reasons, for example, age-related declines in memory and the speed of information processing [26]. We found examples of both models when navigating safely in everyday life and avoiding falls. In addition, the slower process of ‘risk-as-analysis’ also involved a variety of feelings when anticipating the possible outcomes of a planned action. This implies that the two models are interrelated in that cognitive appraisals give rise to emotions and emotions influence appraisals [27].

Both models share resemblances with, and complements, Selection, Optimization, and Compensation [SOC] theory. This action-theoretical model proposes that people make choices that have the greatest personal benefit and relevance to their lives and that these choices are often related to goal setting and goal pursuit [28]. *Selection* is defined as actively or passively reducing the number of activities or goals in order to focus on those areas that are most important in one’s everyday life and are the most preferred [29]. This might mean giving up certain activities in order to focus on activities that are the most important in everyday life, for example, refraining from climbing a ladder because it is perceived as too dangerous when alone. The ageing body and physical impairments were often mentioned among the participants as reasons for adopting more cautious behaviour. *Optimization* is defined as the use of new and alternative means to reach the selected goals and refers to adaptive processes or strategies [29]. For example, lying flat on the floor when vacuuming under the bed or changing from a men’s bike to a women’s bike in order to keep bicycling in spite of poor balance. C*ompensation* refers to methods to compensate for limitations, loss, or decline [29]. One might compensate for impairments by using inherent skills, resources, or external aids such as crutches to compensate for balance impairments or assistive devices such as a mobile phone to be able to call for help when away from home.

SOC theory has been proposed as a model for theory-driven and community-based educational programmes to promote health and to manage long-term interventions such as fall prevention [30]. It has been stressed that comprehensive approaches on different levels should be used when implementing evidence-based interventions because obstacles to change can arise at different stages, including the participant level, the professional level, the organisational level, and the wider environment [31]. In order to enhance the implementation of fall preventive interventions, all levels need to be addressed. Our findings make an important contribution to the planning, development, and implementation of fall prevention programmes. The findings indicate that the awareness about fall risks in everyday life was enhanced by being part of reflective meetings regardless of what level of awareness the participants had from the start. This stresses the value of offering opportunities for older people to discuss falls and fall prevention with peers and professionals in the field who together create a learning culture [32]. Furthermore, in order to improve the conditions to make appropriate judgements in relation to fall risk, a self-management approach might be added that includes skills such as problem-solving and decision-making skills [13].

It has been suggested that, in general, women and men of all ages worry about different risks, judge the magnitudes of risks differently, and differ in their willingness to become involved in health-related activities [33–34]. However, gender differences in relation to risk awareness and risk-taking behaviour tend to decrease with increasing age [35]. This might be true among the participants in our study, and both women and men shared many experiences of fall risk. For example, women and men both admitted to feeling insecure and afraid to fall at times and that they made conscious choices to avoid falls. However, some interesting gender differences were still found in our data. The men often experienced themselves as weak when not being able to perform “masculine” activities that they had always been doing, for example, riding a men’s bicycle or shovelling snow off the roof. Being deprived of such activities might have made them feel like less of a man. Women were in general more sensible about protecting themselves from falls and were concerned about the consequences of a fall. Several of the women had experiences from earlier fractures and were living alone. Women who have suffered fractures might narrow their life space due to the physical injury or to the fear of falling again [36]. Their freedom of movement and independence was important to the participants in this study and they knew that it would be very difficult for them to manage on their own if they suffered from another fracture.

Older people in general have been found to be less prone to engage in risky recreational activities and more likely to attend voluntary annual health check-ups than younger individuals [35]. However, our findings suggest that both older men and women might deliberately expose themselves to situations of obvious fall risk. By ignoring obvious risks of falling they felt great contentment and pride, and increased self-confidence when they managed the situation. These findings are largely in agreement with research on older people’s perceptions of general risks that proposes that some older people might need a certain level of risk exposure to maintain their quality of life [37]. The participants in this study were also in general resourceful when acting to avoid falls either by adapting their movements or by compensating in one way or another, and they reported some innovative ideas. This is in agreement with other studies [10, 38]. Many participants were already participating in exercising individually such as walking with Nordic poles or at the gym, or in exercise programmes such as fitness training or aqua aerobics, on a regular basis, but none of the participants had the specific aim of reducing falls. Several of the participants reported that their adult children looked upon them as old and fragile, and this finding is consistent with other studies showing that older people are often the subjects of paternalistic feelings in which the older person is looked upon as high in warmth but low in competence, so-called affective ageism [39–40]. There might, therefore, be reason to adopt an inter-generational perspective when planning for fall prevention interventions in order to discuss attitudes towards older people. Furthermore, older people might worry that they will be reprimanded and required to restrict their ordinary and highly cherished activities if they attend a fall-prevention programme [10]. However, our findings indicate that older people learn from and share experiences with peers rather than accepting professional advice that might be perceived as patronizing. Routinely distributing lists of suggestions on how to improve safety in the home environment to all older people should, therefore, be avoided.

### Methodological considerations

When designing the study, we used the 32-item COREQ [consolidated criteria for reporting qualitative research] [21] checklist. A strength was the study design with focus group sessions. By meeting with other community-dwelling participants on several occasions, the participants had the opportunity to reflect about fall risks in their everyday lives, both at the sessions and between the sessions, and this resulted in rich data material. Our sampling strategy based on previous experiences resulted in a sufficient variation in gender, history of falls and living arrangements. In contrast, there was little variation in work experiences because most participants were retired white-collar workers and were mainly resource-rich, well educated, and had previous occupational experiences. This limits the transferability of the study findings, and they might not be applicable, for example, to frail older people living in nursing homes. Another strength was that the researchers involved were from different fields of research, and this enriched the way we collected and analysed the data [41]. Approaching practice from theoretical fields that involve a diversity of experiences, for example, gender and physiotherapy studies, contributes to creativity in the analysis. The interpretation of the text was validated through a back-and-forth analysis of the parts (e.g., categories) and the whole transcriptions. The risk that the participants did not feel completely free to express themselves because of the number of researchers involved was recognized and discussed within the research group, and encouraging strategies were taken. The emphasis on appreciation in the PAAR methodology might have influenced the participants to mainly describe positive events and experiences, but negative experiences were also described. The focus group sessions were held during the winter, so many of the experiences concerned winter activities and this limits transferability to countries with less snowy and icy winters.

## Conclusions

Based on the results it may be concluded that the fall risk awareness process might be initiated for various reasons and can involve different feelings and precautions as well as different strategies. This finding highlights that there are many possible channels to reach older people with information about fall risk and fall prevention, including the media and their peers. The findings of this study offer a deeper understanding of older peoples’ conceptualizations about fall risk awareness and make an important contribution to the development and implementation of fall prevention programmes.
